# Synergistic Degradation of a Hyperuricemia-Causing Metabolite Using One-Pot Enzyme-Nanozyme Cascade Reactions

**DOI:** 10.1038/srep44330

**Published:** 2017-03-13

**Authors:** Secheon Jung, Inchan Kwon

**Affiliations:** 1 School of Materials Science and Engineering, Gwangju Institute of Science and Technology (GIST), Gwangju 61005, Republic of Korea

## Abstract

Multi-enzyme cascade reactions are frequently found in living organisms, in particular when an intermediate should be eliminated. Recently, enzyme-mimic nanomaterials (nanozymes) received much attention for various applications, because they are usually more stable and cost-effective than enzymes. However, enzyme-nanozyme cascade reations have not been yet extensively exploited. Therefore, in this study, we investigated one-pot enzyme-nanozyme cascade reactions using urate oxidase (UOX) and catalase-mimic gold nanoparticle nanozyme (AuNP) with the ultimate goal of treatment of hyperuricemia. UOX degrades hyperuricemia-causing uric acid, but also generates hydrogen peroxide raising several health concerns. We successfully demonstrated that one-pot UOX-AuNP cascade systems degrade uric acid more than five times faster than UOX alone, by eliminating potentially cytotoxic hydrogen peroxide, similar to enzyme-enzyme reactions.

Hyperuricemia is a phenomenon marked by an abnormally high level of uric acid in the blood. Such a high level of uric acid may precipitate in certain tissues, associated with cardiovascular diseases, kidney diseases, gout and tumor lysis syndrome (TLS)^1^,^2^,^3^,^4^,^5^,^6^,^7^. TLS is a potentially life-threatening disorder followed by systemic chemotherapy. Gout is a form of inflammatory arthritis caused by uric acid crystal formation^8^. Over eight million people suffer from gout in the US^9^. One way to treat hyperuricemia is to enzymatically degrade uric acid. Urate oxidase enzyme (UOX) catalyzes the conversion of insoluble uric acid into soluble 5-hydroxyisourate and hydrogen peroxide (Fig. 1)^10^. Rasburicase, a recombinant UOX originally derived from *Aspergillus flavus*, was approved by the Food and Drug Administration (FDA) for the treatment of TLS. Rasburicase showed a higher efficacy in decreasing plasma uric acid compared to a conventional chemical drug, allopurinol^11^. Pegloticase, a poly(ethylene glycol)-conjugated porcine-like urate oxidase, was approved by the FDA for the treatment of gout. Despite effective treatment of hyperuricemia using UOXs, there are some health concerns about hydrogen peroxide generated during uric acid degradation. First, patients with glucose-6-phosphate dehydrogenase deficiency are vulnerable to hydrogen peroxide generated during the action of rasburicase and pegloticase, leading to methemoglobinemia^12^,^13^,^14^. Second, hydrogen peroxide can be degraded into hydroxyl radicals by Fenton reaction, causing damages to DNA^15^. Besides the health concerns, the accumulation of hydrogen peroxide slows down the degradation of uric acid. Therefore, elimination of hydrogen peroxide generated during uric acid degradation is considered a promising strategy for mitigating hydrogen peroxide-associated health concerns and accelerating uric acid degradation.

Catalase is an enzyme that catalyzes the dissociation of hydrogen peroxide into oxygen and water. Although catalase may efficiently eliminate hydrogen peroxide generated during uric acid degradation by UOX, it has several intrinsic limitations as an enzyme, such as low stability and relatively high production cost^16^. In order to overcome these limitations, enzyme-mimic nanoparticles (nanozymes) were developed. So far, various nanozymes have been developed to mimic natural enzymes, including peroxidase, oxidase, superoxide dismutase, and catalase^17^,^18^,^19^,^20^,^21^,^22^,^23^. In particular, the combination of diverse oxidase enzymes and peroxidase-like nanozymes have been utilized for biosensor development detecting various target molecules, such as glucose, galactose, cholesterol, and choline^24^,^25^,^26^,^27^,^28^,^29^,^30^. In contrast to natural enzymes, nanozymes usually have good stability and relatively low production cost^16^. Therefore, we hypothesized that the combined use of UOX and a catalase-mimic nanozyme would efficiently remove hydrogen peroxide eliminating any potential damage to cells and tissues. Furthermore, in UOX-nanozyme cascade reactions, uric acid degradation would be accelerated by the simultaneous removal of hydrogen peroxide, a product of uric acid degradation (Fig. 1).

So far, several catalase-like nanozymes have been reported, including gold nanoparticles (AuNPs) and platinum nanoparticles^19^,^31^,^32^,^33^,^34^. Considering the ultimate therapeutic application of UOX-nanozyme cascade system, we chose AuNPs coated with polyvinylpyrrolidone (PVP). He *et al*. reported both PVP-coated gold nanoparticles exhibit pH-dependent catalytic activity interconversion between SOD and catalase^35^. PVP, generally considered safe, is frequently used as a binder in pharmaceutical pills. PVP has also been utilized as a blood plasma substitute^36^. Considering the good biocompatibility of gold, PVP-coated gold nanoparticles are expected to exhibit very minimal toxicity in humans. In order to evaluate the relative performance of AuNPs coated with PVP, we compared the performance of AuNPs coated with PVP with those with other stabilizing agents including poly ethyleneglycol (PEG), citric acid (CA), and tannic acid (TA).

## Results and Discussion

**Figure 2:**
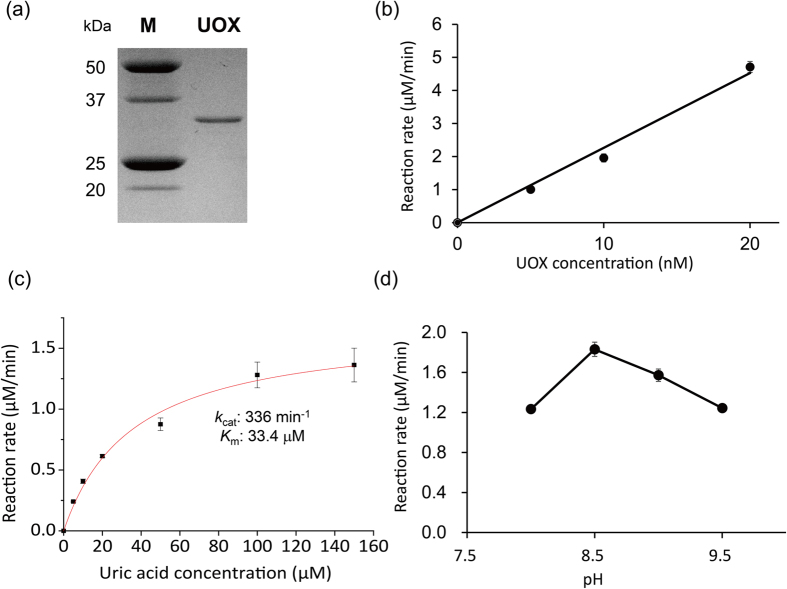
Properties of UOX. (**a**) SDS-PAGE gel image of UOX. M and UOX denote lanes for molecular weight markers and purified UOX, respectively. Full-length gel is presented in Supplementary Fig. S1. (**b**) Reaction rates of at varying UOX concentrations. An appropriate amount of UOX was reacted with 100 μM uric acid in 20 mM borate buffer (pH 9.5). (**c**) Michaelis–Menten plot of UOX. All reactions were conducted in 20 mM borate buffer (pH 9.5) using 5 nM UOX. (**d**) Tendency of UOX activity depending on pH. 100 μM uric acid was converted by 5 nM UOX in 20 nM borate buffer at varying pHs. Standard deviations are expressed by error bars.

In order to construct UOX-nanozyme cascade reaction systems, we first prepared recombinant UOX. Briefly, recombinant UOX was expressed in TOP10 [UOX] *E. coli* expression cells and purified by hexa-histidine tag affinity chromatography. The purified recombinant UOX was visualized by SDS-PAGE (Fig. 2a). The single band was located between 25- and 37-kDa molecular weight standards, which is consistent with the expected molecular weight of UOX, 33.4 kDa. Next, we investigated the kinetics of uric acid degradation by UOX. UOX catalyzes the conversion of uric acid into 5-hydroxyisourate and hydrogen peroxide. Uric acid has absorption at 293 nm. Therefore, initial reaction rates of uric acid degradation by UOX were obtained by monitoring absorbance at 293 nm at varying UOX concentrations (0 to 20 nM) in 20 mM borate buffer (pH 9.5). In the presence of 100 μM uric acid, the initial uric acid degradation rate by UOX linearly increased, as UOX concentration increased up to 20 nM (Fig. 2b). The initial uric acid degradation rates by 5 nM UOX at varying uric acid concentrations (0 to 150 μM) were obtained in 20 mM borate buffer (pH 9.5) in triplicates (Fig. 2c). The average uric acid degradation rates were fitted to a Michaelis-Menten model in order to determine kinetic parameters. The *k*_cat_ and *K*_m_ values of UOX were 336 min^−1^ and 33.4 μM, respectively. The catalytic activity profile of UOX was determined at 5 nM UOX and 100 μM uric acid over a pH range of 8.0 to 9.5 (Fig. 2d). The maximum catalytic activity of UOX was observed at pH 8.5. These results clearly indicated that the purified recombinant UOX efficiently degrades uric acid. However, it was expected that potentially toxic hydrogen peroxide as well as 5-hydroxyisourate was generated upon degradation of uric acid by UOX. Furthermore, the generated hydrogen peroxide would inhibit UOX activity for decomposition of uric acid. We experimentally confirmed that hydrogen peroxide exhibits product inhibition to UOX activity (Supplementary Information Fig. S2).

In order to prevent the accumulation of hydrogen peroxide, we investigated the properties of catalase-mimic nanozymes. First, we used 5 nm AuNPs coated with PVP (Au@PVP), which was reported to have catalase-like activity under basic conditions^35^, in order to achieve cascade reactions with UOX. If not mentioned otherwise in this manuscript, Au@PVP means the Au@PVP with the average size of 5 nm. The TEM image of Au@PVP showed that the average size of Au@PVP is around 5 nm, as expected (Supplementary Information Fig. S3). Since UOX also exhibited significant catalytic activities under basic conditions (Fig. 2d), we performed cascade reactions to degrade uric acid using UOX and AuNPs under basic conditions.

**Figure 3:**
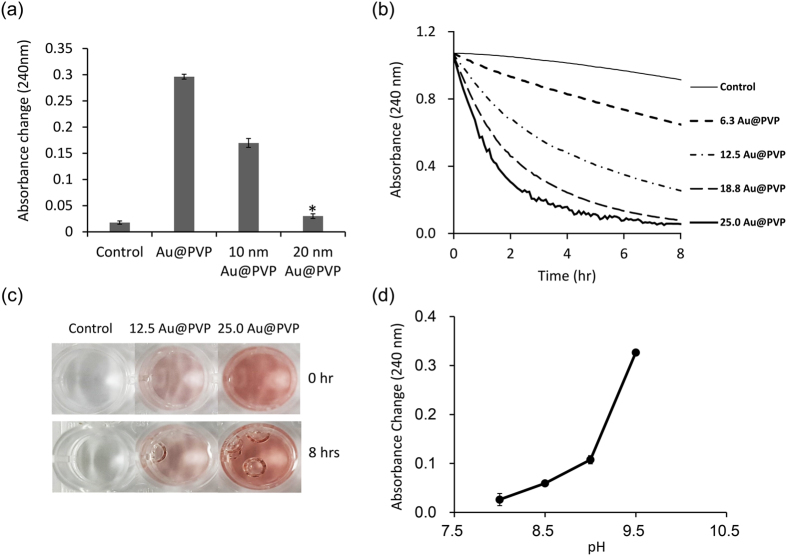
Properties of AuNPs coated with PVP. (**a**) Decomposition of hydrogen peroxide by different sizes of Au@PVP (12.5 μg/mL). Degradation of hydrogen peroxide (25 mM) was monitored by measuring absorbance at 240 nm in 20 mM borate buffer (pH 9.5) for 3 hrs. *Indicates p < 0.01 versus Control (two-tailed Student’s t test). (**b**) Hydrogen peroxide degradation with time at varying amounts of Au@PVP. (**c**) Generation of oxygen bubbles as a reaction product. (**d**) Hydrogen peroxide (25 mM) degradation by Au@PVP for 3 hrs at varying pHs. Standard deviations are expressed by error bars.

Next, we investigated the effects of AuNP size on the catalytic activities of AuNPs. As the size of Au@PVP became smaller, the catalase-like activity of Au@PVP increased (Fig. 3a). We speculate that the smaller AuNPs have relatively larger surface area-to-volume ratios making them catalytically more efficient. As hydrogen peroxide was dissociated to oxygen and water in aqueous solution, oxygen bubbles were generated. In the absence of Au@PVP, no significant oxygen bubble formation was observed (Fig. 3c). However, in the presence of 12.5 μg/mL Au@PVP, oxygen bubble formation was clearly observed after 8 hours (Fig. 3c). As the amount of Au@PVP increased to 25.0 μg/mL, more bubble formation was observed (Fig. 3c). In order for us to observe bubble formation, the 96-well plate containing samples were not shaken during the dissociation experiments. However, in order for us to obtain the time course of hydrogen peroxide dissociation with different amounts of Au@PVP, 0, 6.3, 12.5, 18.8, and 25.0 μg/mL, respectively, the 96-well plate containing samples were shaken for 30 secs for each measurement to reduce oxygen bubble formation.

Decomposition of hydrogen peroxide was monitored by absorbance change at 240 nm. The time courses of hydrogen peroxide dissociation are shown in Fig. 3b. Hydrogen peroxide was decomposed by itself under basic condition, but the rate was very low compared to those in the presence of Au@PVP (Fig. 3b). The time course curve of hydrogen peroxide dissociation in the presence of 25.0 μg/mL Au@PVP slightly fluctuated, likely due to some oxygen bubbles generated by too fast decomposition of hydrogen peroxide (Fig. 3b). However, below 25.0 μg/mL of Au@PVP such as fluctuation in the absorbance time course was not observed Au@PVP, probably due to removal of oxygen bubbles by shaking. Next, we compared the amounts of hydrogen peroxide dissociated by Au@PVP for 3 hours at different pHs (8.0, 8.5, 9.0, and 9.5). As the pH increased from 8 to 9,5, the amount of hydrogen peroxide dissociated increased more than 12 times (Fig. 3d); this trend is consistent with the results reported previously^35^. In order to compare the catalase-activity of AuNPs with that of catalase enzyme, we determined turnover frequency of Au@PVPs by plotting reaction rate vs Au@PVP concentration data (Supplementary Information Fig. S4). When turnover frequency is defined by number of hydrogen peroxide molecules decomposed by a single Au@PVP particle for a given time, the turnover frequency of Au@PVP (5 nm) was 98 sec^−1^. The turnover number of catalase enzyme is 4 × 10^7^ sec^−1^ 37. Although the turnover number of catalase is quite greater than the turnover frequency of Au@PVP, catalase loses its catalytic activity shortly due to the action of its suicide substrate, hydrogen peroxide^38^. Time courses of hydrogen peroxide decomposition by catalase and Au@PVP are presented in Supplementary Information Fig. S5. As expected, the catalase activity significantly dropped within 30 mins, whereas Au@PVP exhibited the almost fully retained activity at least up to 3 hrs.

**Figure 4:**
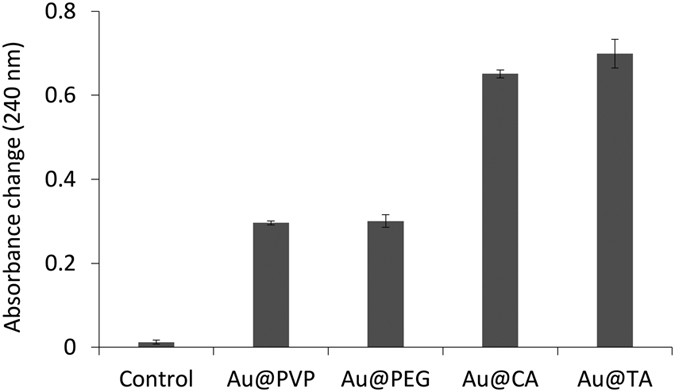
Comparison of catalytic activities of 5 μg/mL AuNPs coated with different stabilizing agents. Monitoring the hydrogen peroxide (25 mM) degradation by various AuNPs (12.5 μg/mL) was proceeded in 20 mM borate buffer (pH 9.5) for 3 hrs. Standard deviations are expressed by error bars.

Next, we investigated the effects of AuNP stabilizing agents on their catalase-like activities. We compared the catalytic activity of AuNPs coated with PVP, PEG, CA and TA (Fig. 4). The amounts of degraded hydrogen peroxide by Au@CAs and Au@TAs were about two times greater than those by Au@PVPs and Au@PEGs (Fig. 4). We speculate that the scavenging activity of hydrogen peroxide/hydroxyl radical of TA and CA is attributed to the enhanced hydrogen peroxide degrading activities of Au@CAs and Au@TAs. It was reported that TA and CA have a very strong scavenging activity of hydrogen peroxide and hydroxyl radicals, respectively^39^,^40^.

Based on catalytic activities of UOX and AuNP nanozymes, we performed UOX-AuNP nanozyme cascade reactions to degrade uric acid. In UOX-AuNP nanozyme cascade reactions, UOX converts uric acid into 5-hydorxyisourate and hydrogen peroxide followed by dissociation of hydrogen peroxide into oxygen and water by AuNP nanozymes. Therefore, we hypothesized that continuous removal of hydrogen peroxide, an intermediate, by AuNP nanozymes will promote uric acid degradation by UOX. In the absence and in the presence of AuNP nanozymes, uric acid degradation by 5 nM UOX was performed in 20 mM borate buffer (pH 9.5). Uric acid degradation was monitored by measuring absorbance at 293 nm.

**Figure 5:**
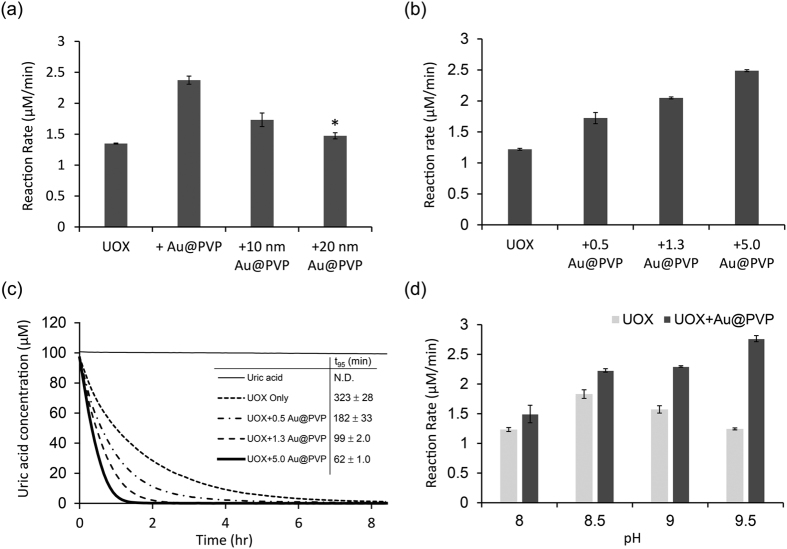
Cascade reactions with UOX and Au@PVP. (**a**) The reaction rate of UOX with different sizes of Au@PVPs (5.0 μg/mL). The measurement of 5 nM UOX reaction rate was performed at 20 mM borate buffer (pH 9.5) with 100 μM uric acid. *Indicates p < 0.05 versus UOX (two-tailed Student’s t test). (**b**) Degradation rates of 100 μM uric acid by 5 nM UOX alone or 5 nM UOX plus varying amounts of Au@PVP in 20 mM borate buffer (pH 9.5). (**c**) Time courses of 100 μM uric acid degradation by 5 nM UOX alone or 5 nM UOX plus varying amounts of Au@PVP in 20 mM borate buffer (pH 9.5). (**d**) Degradation rates of 100 μM uric acid by 5 nM UOX alone or 5 nM UOX plus 5.0 μg/mL Au@PVP in 20 mM borate buffer at varying pHs. Error bars represent standard deviations.

First, it was investigated that the performance of UOX-AuNPs cascade system depending on the size of Au@PVPs. As the size of Au@PVPs decreased, the UOX activity increased (Fig. 5a). These results can be attributed to the fact that smaller particles have relatively larger surface area-to-volume ratios making them catalytically more efficient (Fig. 3a).

Next, the activity UOX with different amount of Au@PVP was examined. When the amount of Au@PVP nanozymes increased, the degradation rate of uric acid increased (Fig. 5b). In the presence of 5.0 μg/mL of Au@PVP nanozymes, the degradation rate of uric acid was two times faster compared with that without any Au@PVP nanozyme (Fig. 5b). In the absence of Au@PVP nanozymes, the time required for 95% uric acid degradation (t_95_) was 323 mins (Fig. 5c). As expected, in the presence of 0.5, 1.3, or 5.0 μg/mL Au@PVP nanozymes, t_95_ decreased to 182, 99, and 62 mins, respectively (Fig. 5c). UOX with 5 μg/mL Au@PVP nanozymes reached t_95_ more than five times earlier than UOX without Au@PVP nanozyme. In the presence of Au@PVP nanozymes, the accelarated degradation of uric acid was attributed to the degradation of an intermediate, hydrogen peroxide, into oxygen and water and the supplementation of oxygen to uric acid degradation reaction. Next, we investigated whether the effects of Au@PVP nanozyme addition on the uric acid degradation rate are pH-dependent. Among pHs ranging from 8.0 to 9.5, the degradation rate of uric acid by UOX was the highest at pH 8.5 (Fig. 5d). At pH 8.5, the addition of 5.0 μg/mL Au@PVP nanozymes increased the degradation rate of uric acid by 20% (Fig. 5d). Above pH 8.5, as pH increased, degradation rate by UOX gradually decreased. However, at pH 9.0 and 9.5, the addition of 5.0 μg/mL Au@PVP nanozymes increased the degradation rate of uric acid by 46% and 123%, respectively. This increase was attributed to the increased catalytic activity of Au@PVP nanozymes at higher pHs (Fig. 3d). Further, at pH 9.5, the uric acid degradation rate by UOX and Au@PVP nanozymes was the highest. However, we could not exclude the possibility that Au@PVP nanozymes directly degraded uric acid, leading to an increase in the uric acid degradation rate. In order to investigate this, we measured the uric acid degradation rate at varying concentrations of Au@PVP nanozymes. As expected, the uric acid degradation by 6.25 or 12.5 μg/mL Au@PVP nanozymes for 3 hrs was only 0.57 or 0.03% of that by 5 nM UOX, respectively (Supplementary Information Fig. S6). Therefore, we concluded that the direct degradation of uric acid by Au@PVP can be ignored compared to that by UOX. Last, we investigated whether Au@PVP substantially aggregate during the uric acid degradation reaction. Aggregation of Au@PVP significantly reduces the total surface area of Au@PVP, very likely leading to a significant reduction in catalytic activities. When Au@PVP aggregate, there is a red-shift in surface plasmon resonance (SPR) band (about 520 nm)^41^,^42^,^43^. Au@PVP with or without UOX were incubated with 100 μM uric acid for 1 hr, and then subjected to UV-visible spectrum analysis. The SPR band of Au@PVP was not significantly changed even in the presence of UOX and after the 1 hr-cascade reaction (Supplementary Information Fig. S7). These results clearly indicated that there was no significant aggregation even after the cascade reaction.

**Figure 6:**
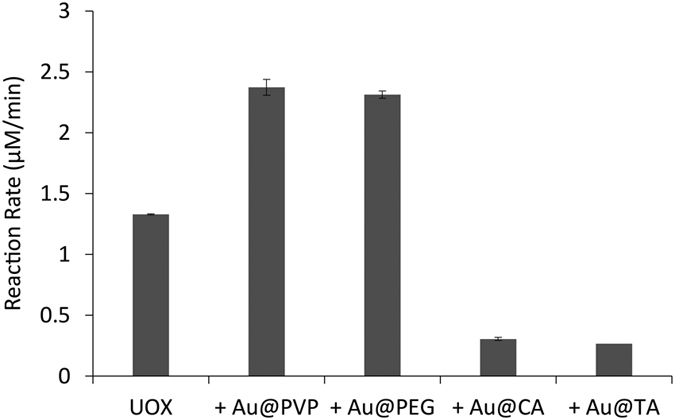
Cascade reaction of UOX and AuNPs depending on different AuNPs capping agents. 100 μM uric acid degradation rate by 5 nM UOX alone or UOX with 5.0 μg/mL AuNPs coated with different capping agents in 20 mM borate buffer (pH 9.5).

Last, we investigated the uric acid degradation rates in the cascade systems consisting of UOX and AuNPs coated with varying stabilizing agents. The effects of stabilizing agents on the UOX-AuNP cascade reaction rate were different from ones on the hydrogen peroxide degradation rate (Fig. 6). The addition of AuNPs coated with PVP (Au@PVP) or PEG (Au@PEG) increased the uric acid degradation rate by 78% or 73%, respectively. However, the addition of Au@CA or Au@TA reduced the uric acid degradation rate by about 23 or 20%, respectively, though both Au@CA and Au@TA exhibited greater hydrogen peroxide degradation rates than Au@PVP and Au@PEG (Fig. 4). It was reported that a hexahistidine tag (His-tag) of protein binds on the surface of AuNPs through metal-histidine coordination^44^,^45^,^46^. When a stabilizing agent is small and weakly binds on the surface of AuNPs such as citrate, a protein with a His-tag easily binds to Au surface^46^. Such a binding on the surface of AuNPs would perturb the folded structures and activities of a protein. Therefore, we hypothesize that Au@CA and Au@TA bind more easily to UOX than Au@PVP and Au@PEG, resulting in the reduced UOX catalytic activity probably due to the structural changes.

In order to test whether a His-tag of UOX plays a key role in reducing the catalytic activity probably via mediating binding to AuNPs, we investigated the effects of addition of free histidine on the UOX-AuNP cascade reaction rate. We expected free histidines serve as competitors to His-tags. As 3 μM of free histidines were pre-incubated with Au@CA or Au@TA, the UOX activity was significantly recovered (Supplementary Information Fig. S8). These results suggest that a His-tag of UOX mediates UOX binding to Au@CA or Au@TA causing the reduction in UOX activity.

## Conclusion

In summary, we have demonstrated that one-pot UOX-AuNP nanozyme cascade systems more efficiently degrade uric acid than UOX alone, by eliminating potentially cytotoxic hydrogen peroxide and supplying oxygen to UOX for uric acid degradation. Size and stabilizing agent of AuNP nanozymes affected both the hydrogen peroxide decomposition rate and uric acid degradation rate in the UOX-AuNP cascade system. Because of the great biocompatibility of PVP-coated gold nanoparticle nanozymes, the UOX-AuNP nanozyme cascade systems have great potential for the treatment of TLS and gout. Broadly, the results presented here suggest that catalase-mimic AuNP nanozymes can be used to protect cells and tissues by eliminating toxic hydrogen peroxide, which causes other human diseases.

## Materials and Methods

### Materials

Gold nanoparticles coated with PVP (5 nm, 10 nm, and 20 nm), PEG (5 nm), CA (5 nm), or TA (5 nm) were obtained from nanoComposix Inc. (San Diego, CA). Ni-nitrilotriacetic acid (Ni-NTA) agarose and pQE80 plasmid were purchased from Qiagen (Valencia, CA). Vivaspin centrifugal concentrators with a molecular weight cut-off (MWCO) of 50 kDa were purchased from Sartorius Corporation (Bohemia, NY). PD-10 desalting columns were obtained from GE Health care (Piscataway, NJ). All other chemicals were purchased from Sigma-Aldrich Corporation (St. Louis, MO) unless otherwise stated.

### UOX expression and purification

The expression vector for a recombinant urate oxidase (UOX) originally derived from *Aspergillus flavus* with a hexa-histidine tag, pQE80-UOX, was used (Lim *et al*.^47^). The pQE80-UOX plasmid was transformed into TOP10 *Escherichia coli* cells for the expression of UOX, affording TOP10 [UOX] cells. The pre-cultured TOP10 [UOX] cells were inoculated into a newly made 2xYT medium containing 100 μg/mL ampicillin. Then, the cells were incubated with shaking (220 rpm) at 37 °C, until the optical density (OD) at 600 nm reached 0.5. One mM IPTG was added to the cultured TOP10 [UOX] cells to induce protein expression. After induction for 5 hrs, cells were pelleted by centrifuging at 12,000 rpm for 30 min. Then, cell pellets were resuspended with the lysis buffer (pH 7.5) containing 50 mM sodium phosphate, 0.3 M NaCl and 10 mM imidazole in order to purify UOX. The resuspended cell pellets in lysis buffer were incubated with the lysozyme (200 μg/mL) on ice for 30 min. The cell pellets incubated with lysozyme were sonicated on ice for 10 mins (10 sec pulse on and 20 sec pulse off). The cell lysate was pelleted by centrifugation at 12,000 rpm at 4 °C for 30 mins and the supernatant was transferred to a fresh tube. Ni-NTA agarose was mixed with the supernatant for 1 hr with shaking at 220 rpm. The supernatant mixed with the Ni-NTA agarose was loaded on column and washed with washing buffer (pH 7.5) containing 50 mM sodium phosphate, 0.3 M NaCl and 20 mM imidazole. Elution of proteins was performed by elution buffer (pH 7.5) containing 50 mM sodium phosphate, 0.3 M NaCl and 250 mM imidazole. The buffer of protein solution was exchanged with PBS buffer (pH 7.4) by PD-10 column. The molar absorption coefficient of UOX at 280 nm was reported as 53,400 M^−1^ cm^−1^ 47. The protein concentration was determined according to the Beer-Lambert law by measuring molar absorbance at 280 nm using a Synergy H1 four multimode microplate reader (BioTek, Winooski, VT)^48^.

### Enzymatic activity assay of UOX

The kinetic analysis of UOX was performed by the spectrophotometric method. UOX enzymatic reaction, where uric acid is oxidized into 5-hydroxyisourate (5HIU), was determined by monitoring the absorbance reduction at 293 nm in triplicates at 25 °C in a standard 96-well plate on the Synergy™ four multimode microplate reader (BioTek, Winooski, VT). The reaction rate of uric acid (μM/min) was obtained by dividing the rate of OD change (min^−1^) using molar absorptivity of uric acid (12,300 M^−1^ cm^−1^)^49^. The kinetic parameters of UOX at 5 nM was measured in 20 mM borate buffer (pH 9.5) at varying concentrations of uric acid. A Michaelis-Menten model was applied to average consumption rates at each uric acid concentration in order to obtain *V*_max_, *K*_m_ and *k*_cat_.

### Transmission electron microscopy (TEM)

The TEM (JEOL, JEM-2100, Peabody, MA) was used for the morphological and size analysis of 5 nm gold nanoparticles coated with PVP (Au@PVP). A drop of Au@PVP was applied onto 200-mesh carbon-coated copper grids and dried in vacuum oven at 25 °C. The TEM images of Au@PVPs were analyzed using the ImageJ software to determine particles size-distribution.

### Catalytic activity assay of AuNPs

The catalytic activities of AuNPs were spectrophotometrically determined. Hydrogen peroxide was prepared freshly before measurement. The reduction in absorbance at 240 nm caused by degradation of hydrogen peroxide was measured in triplicates at 25 °C by the Synergy H1 four multimode microplate reader. Prior to every measurement, the plate was shaken for 30 secs in order to remove oxygen bubbles generated upon hydrogen peroxide degradation. The absorbance of hydrogen peroxide during the reaction was used as a blank.

### Cascade reactions of uric acid degradation using UOX and AuNPs

The cascade reactions of uric acid degradation were initiated by adding uric acid to the reaction buffer containing UOX and AuNP in 20 mM borate buffer. The reaction was traced by monitoring the absorbance at 293 nm at 25 °C in triplicates.

## Additional Information

**How to cite this article:** Jung, S. and Kwon, I. Synergistic Degradation of a Hyperuricemia-Causing Metabolite Using One-Pot Enzyme-Nanozyme Cascade Reactions. *Sci. Rep.* **7**, 44330; doi: 10.1038/srep44330 (2017).

**Publisher's note:** Springer Nature remains neutral with regard to jurisdictional claims in published maps and institutional affiliations.

## Supplementary Material

Supplementary Information

## Figures and Tables

**Figure 1:**
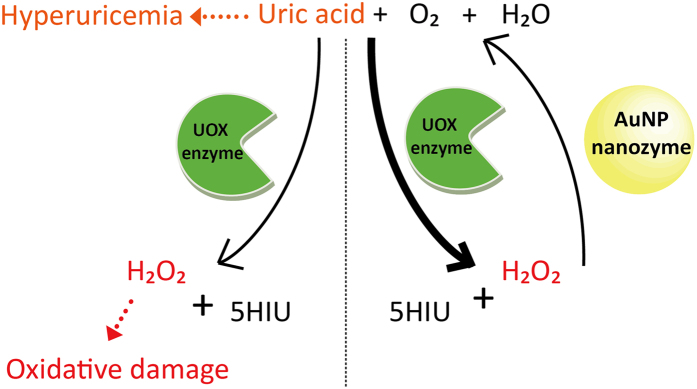
Hyperuricemia-causing uric acid can be degraded into 5-hydroxyisourate (5HIU) and hydrogen peroxide (H_2_O_2_) by urate oxidase (UOX) enzyme in the presence of oxygen and water (left side). A potentially toxic hydrogen peroxide can be dissociated into oxygen and water by AuNP nanozyme (right side). In the presence of both UOX enzyme and AuNP nanozyme, uric acid degradation increases.
