# Supplementary material for: Synergistic Degradation of a Hyperuricemia-Causing Metabolite Using One-Pot Enzyme-Nanozyme Cascade Reactions
Source: Sci Rep. 2017 Mar 13;7:44330. doi: 10.1038/srep44330 (PMC5347090; doi:10.1038/srep44330)
Supplement: Supplementary Information [file srep44330-s1.pdf]

Synergistic Degradation of a Hyperuricemia-Causing Metabolite Using One-Pot Enzyme-Nanozyme Cascade Reactions

Secheon Jung[a] and Inchan Kwon[a,*]

[a]School of Materials Science and Engineering, Gwangju Institute of Science and Technology (GIST), Gwangju 61005, Republic of Korea

[*]Correspondence:

Inchan Kwon

Associate Professor
Gwangju Institute of Science and Technology
School of Materials Science and Engineering
Gwangju, 61005, Republic of Korea
Phone: +82 62-715-2312
Fax: +82 62-715-2304
E-mail: inchan@gist.ac.kr

Supplementary Information

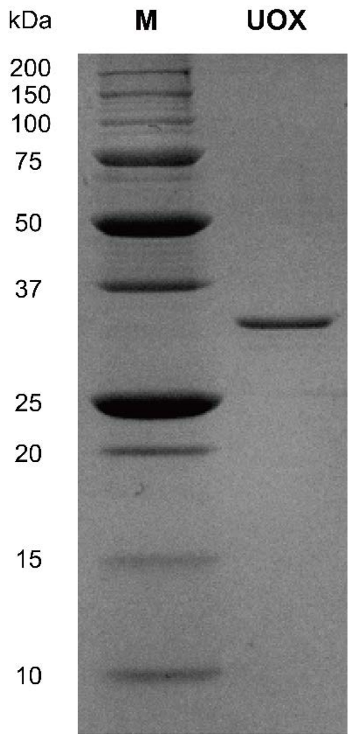

Fig. S1 Full-length SDS-PAGE gel image of UOX. **M** and **UOX** denote lanes for molecular weight markers and purified UOX, respectively.

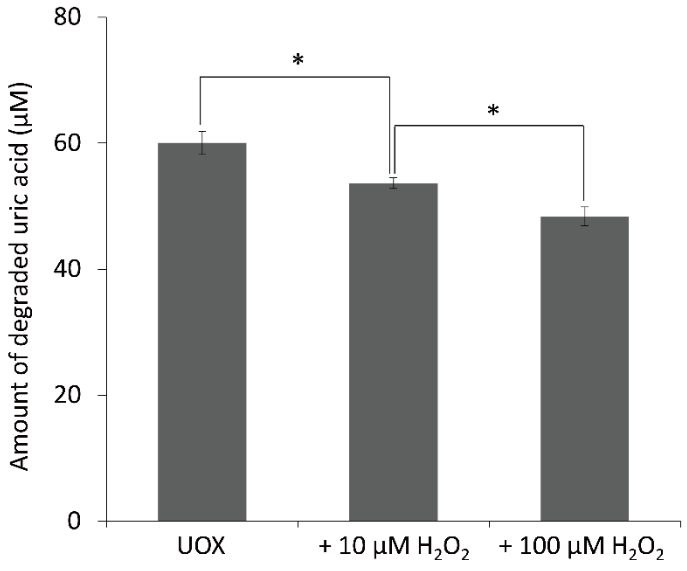

Fig. S2 H_2O_2 inhibition to UOX. The amount of uric acid degraded by 5 nM UOX in pH 9.5 borate buffer for 1h was measured. * indicates $p < 0.05$ (two-tailed Student's t test).

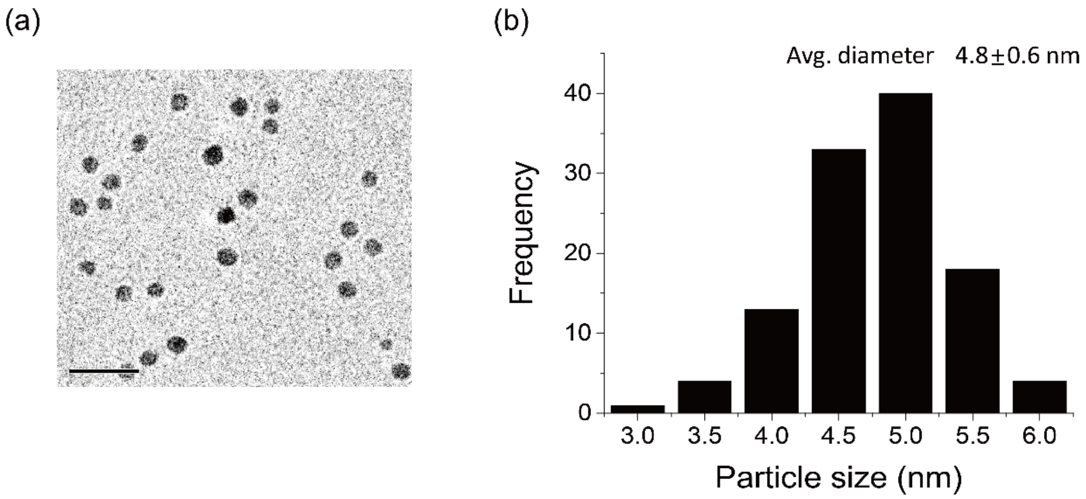

Fig. S3 Physical characteristics of Au@PVP (a) TEM image of Au@PVP with a scale bar of 20 nm (b) Size distribution of AuNP was obtained by TEM image of Au@PVP analyzed with the ImageJ software.

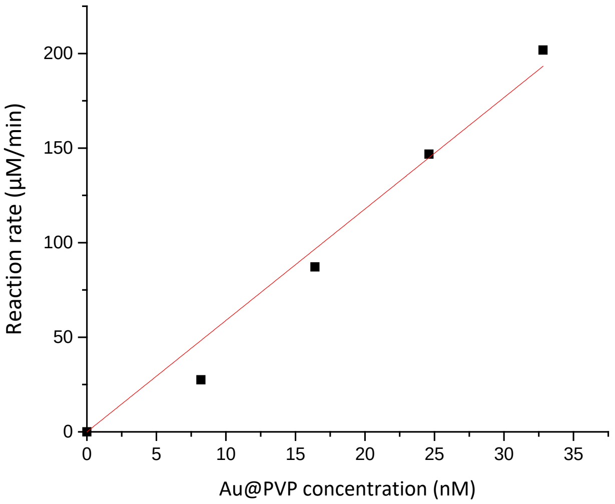

Fig. S4 Hydrogen peroxide degradation rate vs concentration of Au@PVPs for determination of the turnover frequency of Au@PVPs. Hydrogen peroxide (25 mM) was degraded by varying concentrations of Au@PVPs. Au@PVP concentrations were calculated using the method previously reported (Liu, X. *et al.*, *Colloids and Surfaces B: Biointerfaces* 58, 3-7, 2007).

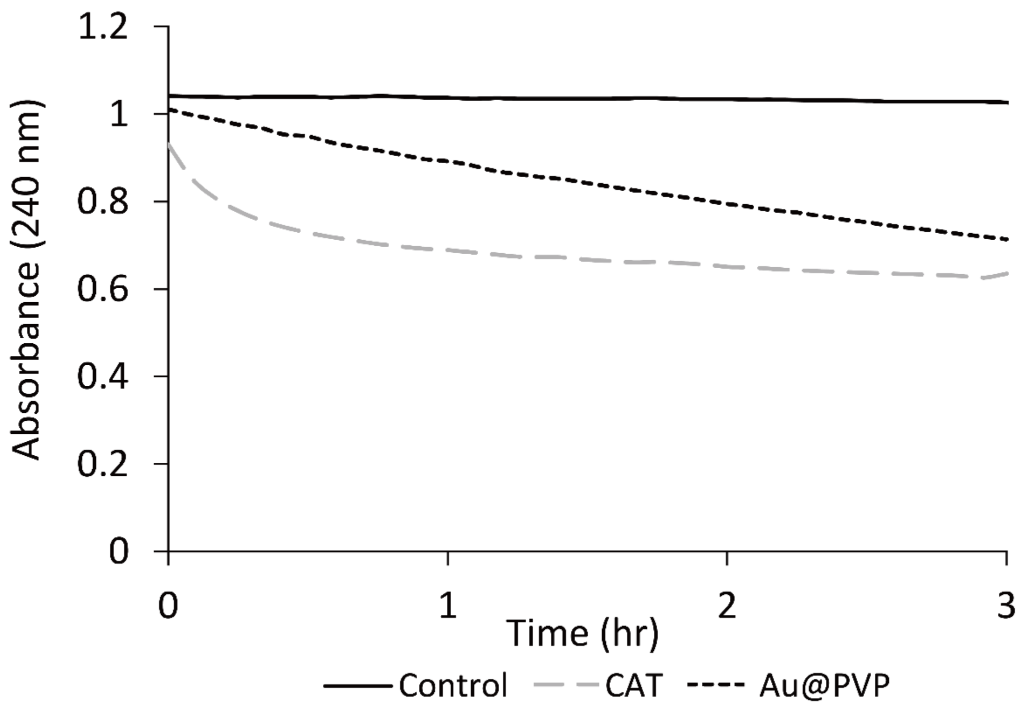

Fig. S5 Comparison of hydrogen peroxide degradation (25 mM) with time without (Control) or with 0.5 μg/mL catalase (CAT) or 12.5 μg/mL Au@PVP.

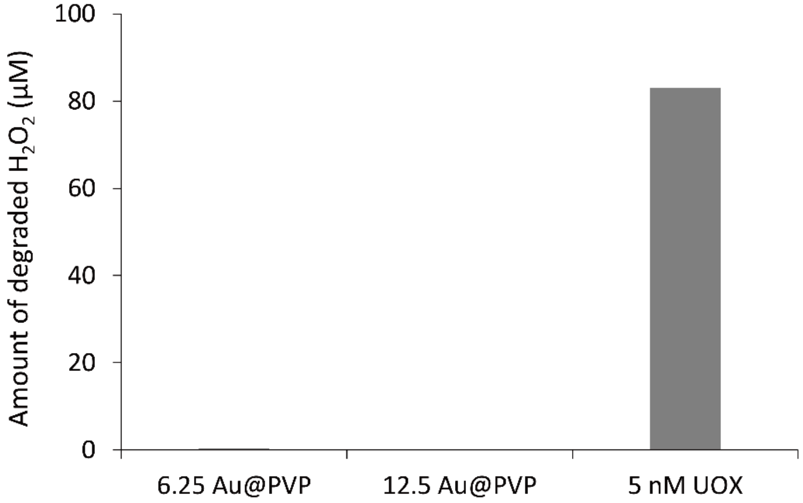

Fig. S6 Uric acid degradation in the presence of 6.25 or 12.5 μg/mL Au@PVP for 3 hrs in 20 mM borate buffer (pH 9.5) compared with that in the presence of 5 nM UOX.

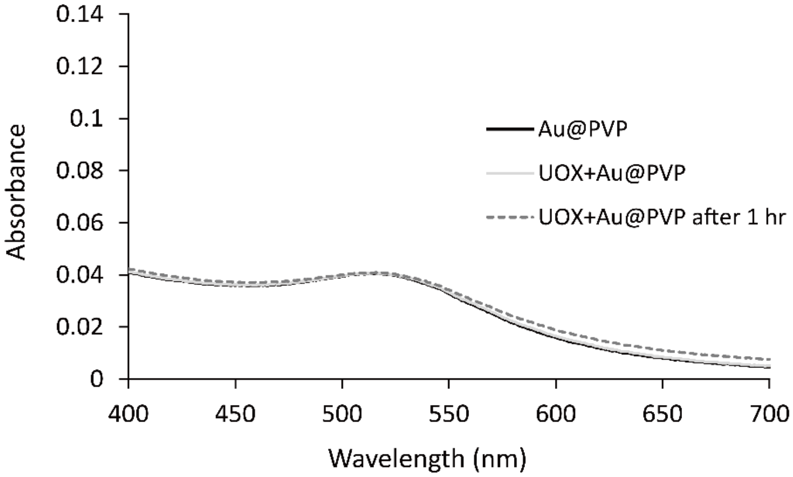

Fig. S7 Absorption spectra of Au@PVP before and after the reaction in 20 mM borate buffer (pH 9.5). UOX (5 nM) and Au@PVP (5.0 μg/mL) were used.

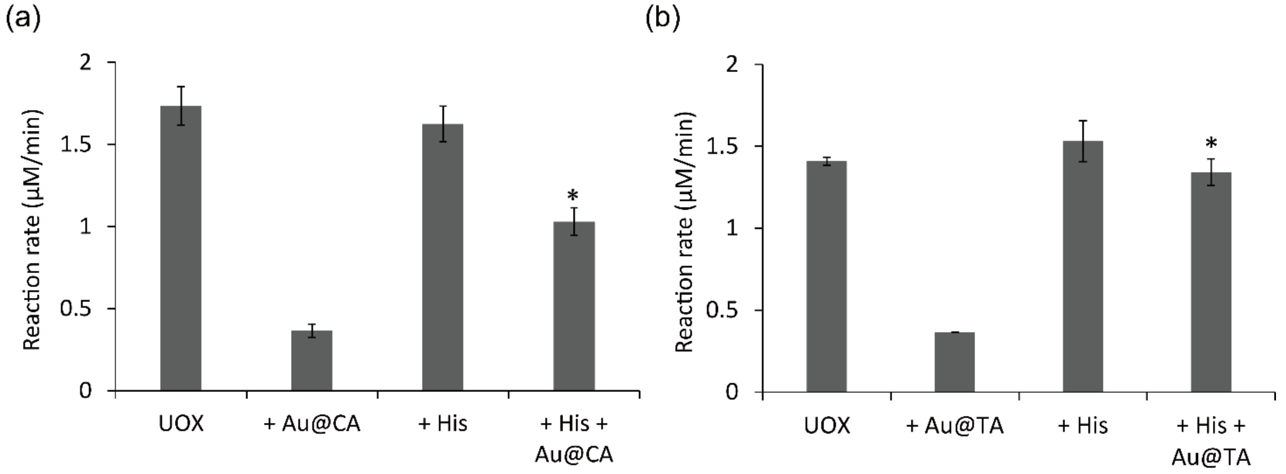

Fig. S8 Effects of free histidine addition on the UOX-Au@CA (a) or Au@TA (b) cascade reaction rate. After 3 μM of free histidines were pre-incubated for 5 mins with 5 μg/mL Au@CA or Au@TA, the mixtures were incubated with 5 nM UOX and 100 μM uric acid in 20 mM borate buffer (pH 9.5). * indicates $p < 0.005$ versus + Au@CA or + Au@TA, respectively (two-tailed Student's t test).
